# Pacific Walrus and climate change: observations and predictions

**DOI:** 10.1002/ece3.381

**Published:** 2012-09-11

**Authors:** James G MacCracken

On page 2087, we missed adding a disclosure statement in the Acknowledgments that “The findings and conclusions in this article are those of the authors and do not necessarily represent the views of the U.S. Fish and Wildlife Service.” We regret this oversight.

The Acknowledgments paragraph should read like the following:
